# Revision of the birch-associated genus *Massalongia* (Diptera, Cecidomyiidae), with description of a new species from Japan and a taxonomic key to worldwide species

**DOI:** 10.3897/zookeys.958.54300

**Published:** 2020-08-11

**Authors:** Ayman Khamis Elsayed, Marcela Skuhravá, Kazuki Ohta, Satoshi Yoshida, Makoto Tokuda

**Affiliations:** 1 The Botanical Gardens, Graduate School of Science, The University of Tokyo, Tokyo 112–0001, Japan The University of Tokyo Tokyo Japan; 2 Department of Applied Entomology, Faculty of Agriculture, Alexandria University, Alexandria, Egypt Alexandria University Alexandria Egypt; 3 Bítovská 1227, Praha 4, Czech Republic Unaffiliated Prague Czech Republic; 4 Laboratory of Systems Ecology, Faculty of Agriculture, Saga University, Saga 840–8502, Japan Saga University Saga Japan; 5 The United Graduate School of Agricultural Sciences, Kagoshima University, Kagoshima 890–0065, Japan Kagoshima University Kagoshima Japan

**Keywords:** *
Betula
*, Betulaceae, Cecidomyiidi, cocoon, DNA barcode, gall midges

## Abstract

*Betula* (Betulaceae), or birch, is a Holarctic genus of trees and shrubs whose species have ornamental, industrial, and medical importance. Gall midges of the genus *Massalongia* (Diptera: Cecidomyiidae: Cecidomyiidi) are exclusively associated with birches in the Palearctic region. In 2018, an undescribed *Massalongia* species was discovered forming leaf galls on the midveins of *B. grossa* on Mount Tara, Saga Prefecture, Kyushu, Japan. In this study the species is described as *M. nakamuratetsui* Elsayed & Tokuda, **sp. nov.**, and a DNA barcode provided for it. The other known species of Massalongia are redescribed because the original descriptions are outdated and insufficient. A lectotype is designated for *M. bachmaieri*. In addition, the monotypic genus *Apagodiplosis*, containing *A. papyriferae* associated with *B. papyrifera* in the Nearctic region, is synonymized here under *Massalongia*, resulting in *M. papyriferae***comb. nov.**, rendering *Massalongia* a Holarctic genus with six species. Comparing the sequence data of *M. nakamuratetsui* with all sequences available in The Barcode of Life Data (BOLD) system supports the occurrence of *Massalongia* in the Nearctic region and suggest that more species could be discovered there. *Massalongia* species form leaf or bud galls, and their mature larvae drop to the ground in autumn and overwinter in characteristic waterproof bottle-like cocoons, which is possibly a protective adaptation for pupation in wet and snowy lands. A taxonomic key to all *Massalongia* species is provided.

## Introduction

*Betula* L. (Betulaceae), or birch, is a genus of trees and shrubs broadly distributed in the northern hemisphere, from the sub-tropics to the arctic. *Betula* species are valued by gardeners and landscapers and are commonly planted in urban areas, roadsides, and parks (Shaw et al. 2014). They constitute the most important sources of hardwood in northern Europe and are also used as fuel and the production of tool handles, barrels, toys and musical instruments (Praciak 2013). Some *Betula* species were used in traditional medicine in different regions of the world (Huh et al. 2011; Al-Snafi 2015; Rastogi et al. 2015). Moreover, some birches are used for the production of tea and beer (Svanberg et al. 2012; Praciak 2013). Thus, investigations on insect fauna associated with birches are important for identifying potential pest species.

At least 17 species of phytophagous gall midges (Diptera: Cecidomyiidae: Cecidomyiinae) are known to occur on *Betula* worldwide, including seven species of *Semudobia* Kieffer, 1913a (Lasiopteridi: unplaced to tribe), four species of *Massalongia* Kieffer, 1897 (Cecidomyiidi: unplaced to tribe), two species each of *Anisostephus* Rübsaamen, 1917 (Cecidomyiidi: Cecidomyiini) and *Dasineura* Rondani, 1840 (Lasiopteridi: Dasineurini), and one species each of *Resseliella* Seitner, 1906 and *Apagodiplosis* Gagné, 1973 (Cecidomyiidi: unplaced to tribe) (Gagné and Jaschhof 2017). In Japan, three gall midge species are known to occur on *Betula*. Namely, *Semudobia betulae* (Winnertz, 1853), *S. tarda* Roskam, 1977, and *S. skuhravae* Roskam, 1977 (Roskam 1977).

**Figures 1–3.**
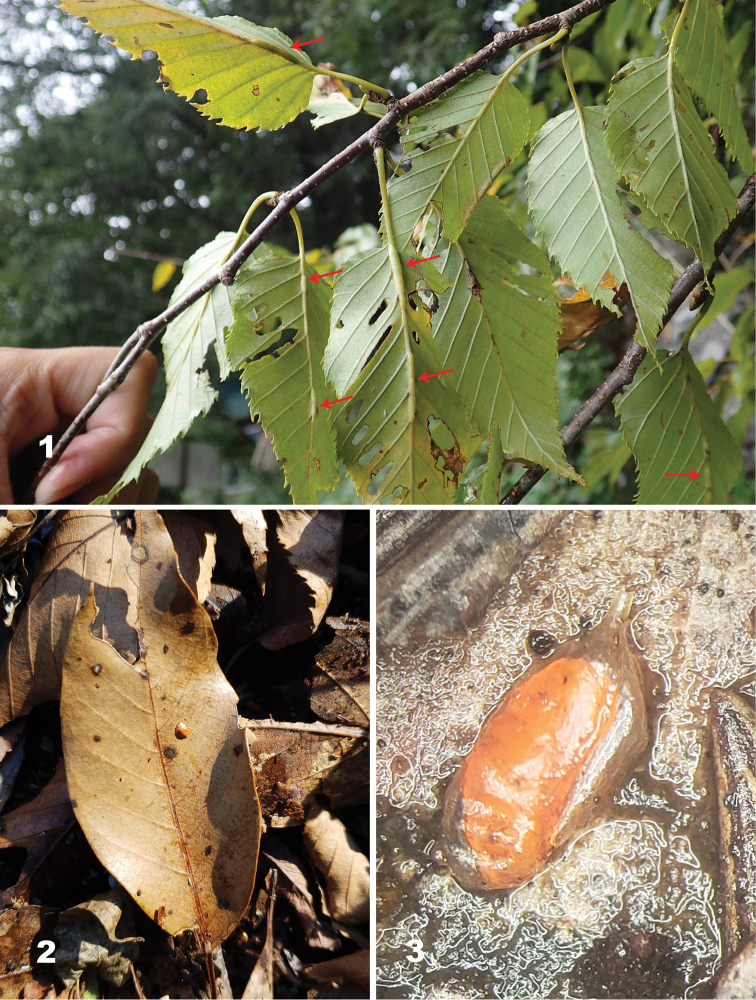
*Massalongia nakamuratetsui* sp. nov. **1** leaf galls (red arrows) on *B. grossa***2** overwintering larva in cocoon on leaf litter **3** bottle-shaped cocoon.

Two of us, K. Ohta and S. Yoshida, discovered leaf galls on *B. grossa* Siebold & Zucc. induced by a gall midge species on Mount Tara, Saga Prefecture, Kyushu, Japan (Fig. 1) during the course of our field investigations. Morphological examinations indicated that the gall midge is an undescribed species of the Palearctic genus *Massalongia*. In this study we review *Massalongia* and describe the Japanese species as new to science. In addition, we synonymize the Nearctic genus *Apagodiplosis* under *Massalongia* because no differences were found between the two genera.

## Materials and methods

*Collecting and rearing*. Leaves with galls on main leaf veins on *B. grossa* (Fig. 1) were collected from Nakayama camp site (elevation 550 m a.s.l.), Mount Tara, Saga Prefecture, Japan (32°59'8"N, 130°5'40"E) in late August and late September 2018. Galls were dissected under a stereoscopic microscope and larvae were preserved in 75% ethanol for morphological examinations and 99.5% ethanol for molecular analysis.

Mature larvae inside their cocoons (Figs 2, 3) were collected from leaf litter under the galled tree in the same location as the earlier leaf collection. Some cocoons were cut open to retrieve larvae and preserve them in 75% ethanol. Remaining cocoons were transferred to plastic cups containing a mixture of peat moss and sand following Elsayed et al. (2018a). The cups were half buried in the soil and maintained until the beginning of March 2019 in a research farm of Faculty of Agriculture, Saga University, Saga Prefecture (elevation 5.5 m a.s.l.). After the cups were brought back to the laboratory, gall midge adults emerged in late March 2019. Adults were preserved in 75% ethanol and pupal exuviae were preserved in 99.5% ethanol.

*Morphological examination and terminology*. Gall midge specimens of the newly described species and *M. bachmaieri* Möhn, 1958 were mounted on microscope slides in Canada balsam following the technique outlined in Gagné (1994), except for the clearing step for the larval and adult specimens following Elsayed et al. (2018b). The slide–mounted specimens were examined under a bright–field and phase–contrast microscope (CX43, Olympus, Tokyo) and line illustrations were made with a mechanical pencil with the aid of a drawing tube. These illustrations were scanned and inked using Apple Pencil 2 and the application Procreate (version 5.0.3) on iPad Pro 2018 (Apple Inc., California). Photomicrographs were taken with a digital camera (DP22, Olympus, Tokyo) attached to a semi-motorized fluorescence microscope (BX53, Olympus, Tokyo).

Morphological terminology mainly follows Kirk-Spriggs and Sinclair (2017) for adults. Larval and pupal terminology follow Gagné (1994). All types of the newly described species are deposited in the collection of Entomological Laboratory, Faculty of Agriculture, Kyushu University, Japan (ELKU).

The ethanol-preserved adults, pupal exuviae and larvae of *M. bachmaieri* were borrowed from the collection of Staatliches Museum für Naturkunde, Stuttgart (SMNS) The holotype and paratypes of *M. betulifolia* Harris, 1974 adults were borrowed from the Natural History Museum in London, United Kingdom (BMNH). The ethanol-preserved larvae of *M. rubra* (Kieffer, 1890) were obtained from the collection of Marcela Skuhravá and mounted on slides following the technique mentioned above.

*DNA extraction*, *sequencing*, *and alignment*. The total DNA was extracted from the whole body of three second instars and one third instar of the Japanese species using the NucleoSpin Tissue kit (Macherey Nagel, Germany) following the manufacturer’s protocol. Fragment of the mitochondrial cytochrome oxidase subunit I (COI) gene was amplified using a TaKaRa Ex Taq (Takara Bio Inc., Shiga, Japan) and following set of primers: J–1718 (5'–GGA GGA TTT GGA AAT TGA TTA GTT CC–3') (Simon et al. 1994) and COIA (5'–CCC GGT AAA ATT AAA ATA TAA ACT TC–3') (Funk et al. 1995). The PCR products were purified using ExoSAP-IT reagent (Affymetrix Inc., USB products, Ohio, USA). The sequencing reaction was performed using the BigDye Terminator Cycle Sequencing Reaction Kit (Applied Biosystems, Foster City, CA, USA). Ethanol precipitation was used for post-reaction cleanup, and an ABI 3130 sequencer (Applied Biosystems) was used for sequence determination. The obtained sequences were aligned using the software MEGA (ver. 6.0) (Tamura et al. 2013), and were deposited in the DNA Data Bank of Japan (DDBJ), European Molecular Biology Laboratory (EMBL), and GenBank (http://www.ncbi.nlm.nih.gov/genbank).

## Results

### Taxonomy

#### Genus Massalongia

Taxon classificationAnimaliaDipteraCecidomyiidae

Kieffer, 1897

1303F17C-109E-5D30-AF2F-F3AC7504B67F

Massalongia Kieffer, 1897: 12. Type species, Hormomyia rubra Kieffer by original designation.
Apagodiplosis Gagné, 1973: 862. Type species, Oligotrophus papyriferae Gagné, comb. nov.

##### Diagnosis.

*Massalongia* differs from other genera of the supertribe Cecidomyiidi in the following combination of characters: antennal flagellomeres are cylindrical in both sexes; male flagellomeres possess three sets of short-looped circumfila that appear interconnected at least in some flagellomeres of each specimen; the reduced abdominal setation; the unmodified female tergite VIII; the presence of dorsal pigmentation on the protrusible part of ovipositor; the massive gonocoxites and mediobasal lobes; the habit of mature larvae to pupate in the soil inside hyaline bottle-shaped cocoons. The following diagnosis lists the attributes shared by known species and can serve as a checklist for future species descriptions.

##### Description.

**Figures 4–11.**
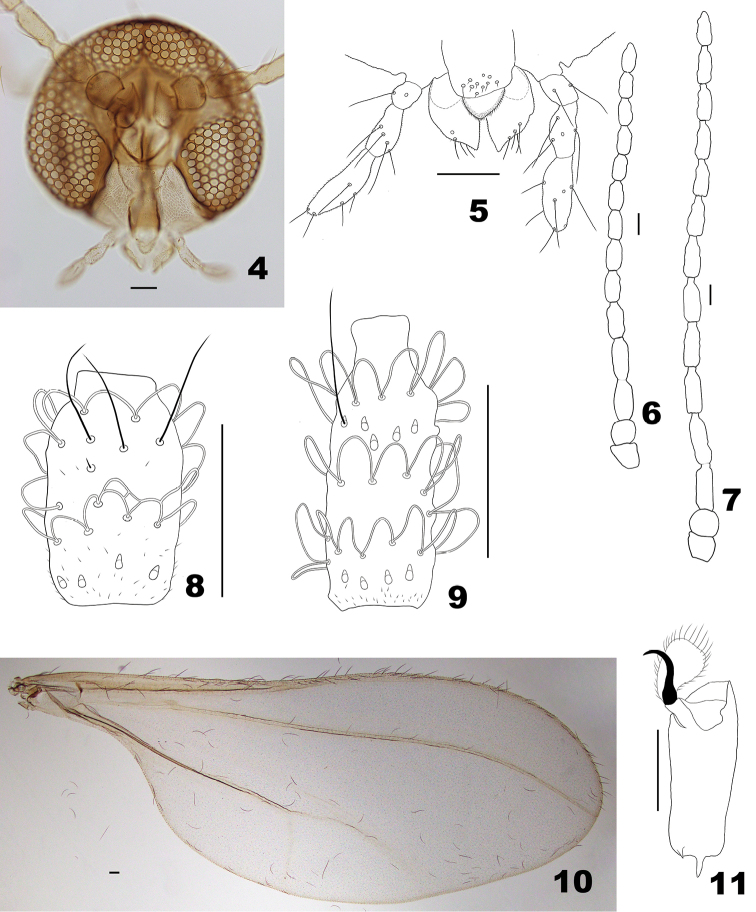
*Massalongia nakamuratetsui* sp. nov. **4** head **5** ventral view of mouthparts **6** female antenna **7** male antenna **8** dorsal view of female flagellomere V. **9** dorsal view of male flagellomere V. **10** wing **11** tarsomere V and acromere. Scale bars: 50 µm.

*Adults. Head*. Eye bridge 5–6 facets long; facets rounded. Occiput without dorsal protuberance (Fig. 4). Mouthparts (Fig. 5): labrum with short setae and no microtrichia; hypopharynx microtrichose; labellum ellipsoid, with stout setae laterally; palpus 3-segmented, consecutively longer, microtrichose, with scattered setae and no scales. Antenna (Figs 6–9): scape conical, pedicel rounded; flagellomeres cylindrical in both sexes, successive flagellomeres diminishing gradually in length; flagellomeres I–II connate. Female flagellomeres with 2 sets of interconnected circumfila. Male flagellomeres with 3 sets of short-looped circumfila, some flagellomeres with interconnected circumfila.

*Thorax* (Figs 10, 11). Wing hyaline; Rs present but rudimentary; R_4+5_ curved toward apex, joining C posteriad of wing apex; C not broken after the conjunction with R_4+5_. Acromere: claws untoothed, bent beyond midlength; empodia longer than claws. Scutum with 4 rows of numerous setae. Anepimeron with setae. Anepisternum and katepisternum bare.

**Figures 12–13.**
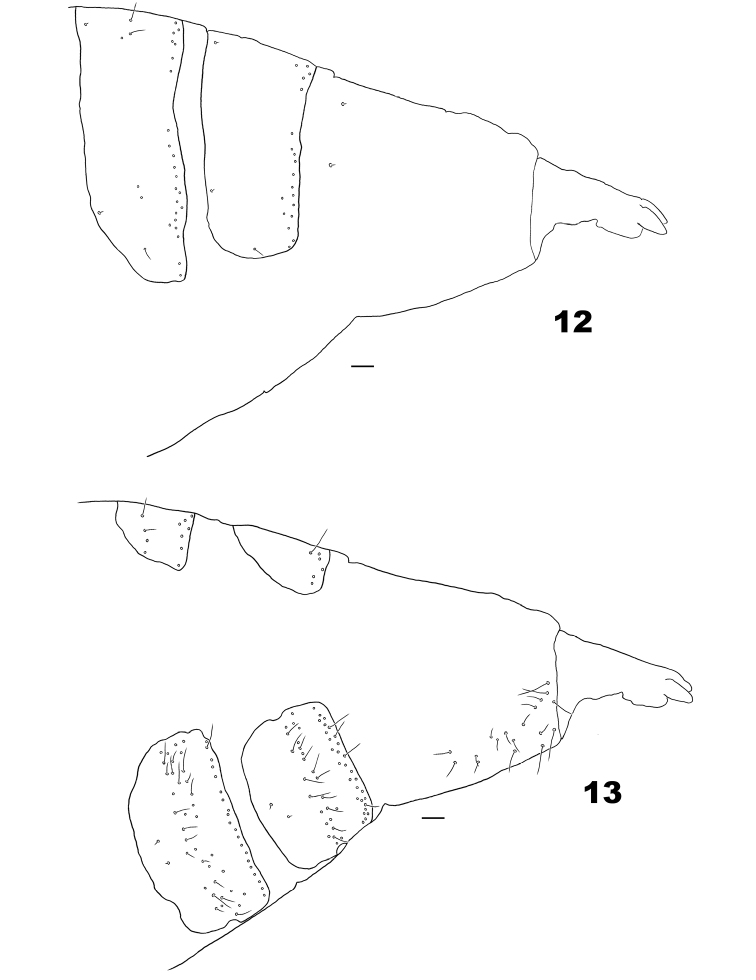
Terminal part of female abdomen of *Massalongia nakamuratetsui* sp. nov. **12** dorso-lateral view **13** ventro-lateral view. Scale bars: 50 µm.

**Figures 14–18.**
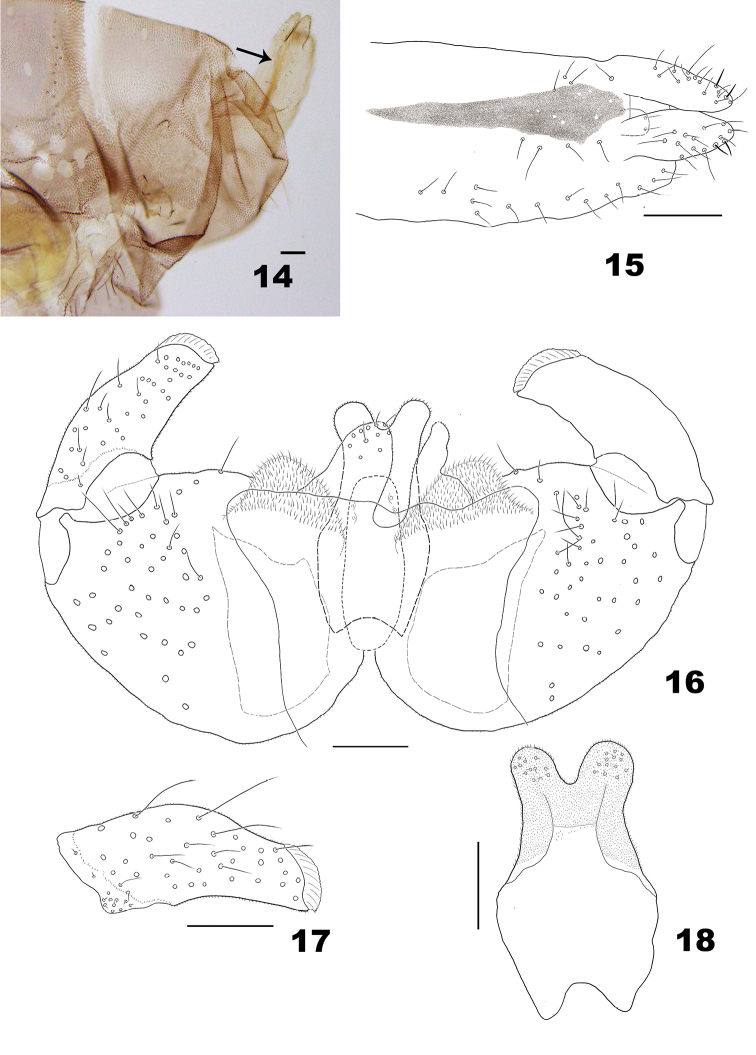
*Massalongia nakamuratetsui* sp. nov. **14** terminal part of female abdomen (arrow indicate the dorsal sclerite on the protrusible portion) **15** protrusible portion of ovipositor **16** male terminalia **17** ventral view of gonostylus **18** ventral view of male hypoproct. Scale bars: 50 µm.

*Female abdomen* (Figs 12–15). Tergites I–VII entire, rectangular, without scales, with anterior pair of trichoid sensilla; tergites I–VI with 1 row of posterior setae; tergite VII with 1–2 rows of posterior setae; tergite VIII unpigmented, differentiated from remainder of tergum only by anterior pair of trichoid sensilla, without scales and setae. Sternites II–VI with scattered setae near midlength, 1 posterior row of setae; sternites III–VII with anteromedial pair of trichoid sensilla; sternite VIII unpigmented, without anterior pair of trichoid sensilla, with scattered setae posteriorly. Ovipositor: protrusible portion with stiff dorsal sclerite, scattered setae ventrally and few setae dorsally; cerci separate, bilaterally flattened, with 2 slightly thickened sensory setae at apex; hypoproct with 2 apical setae.

*Male abdomen*. Tergites I–VII as in female; tergite VIII short, sclerotized only anteriorly, with anterior pair of trichoid sensilla located on the sclerotized part. Sternites II–VI as in female; sternite VII with anteromedial pair of trichoid sensilla, scattered setae near midlength and 1–2 posterior rows of setae; sternite VIII short, with pair of trichoid sensilla placed anterolaterally and 2–3 posterior rows of setae. Terminalia (Figs 16–18): Gonostylus covered mostly with microtrichia and setae dorsally and ventrally, with comb-like denticles; gonocoxite robust, massive, with enlarged mediobasal lobes and microtichose; hypoproct elongate, constricted after midlength, without setae dorsally, with setae posteroventrally.

**Figures 19–21.**
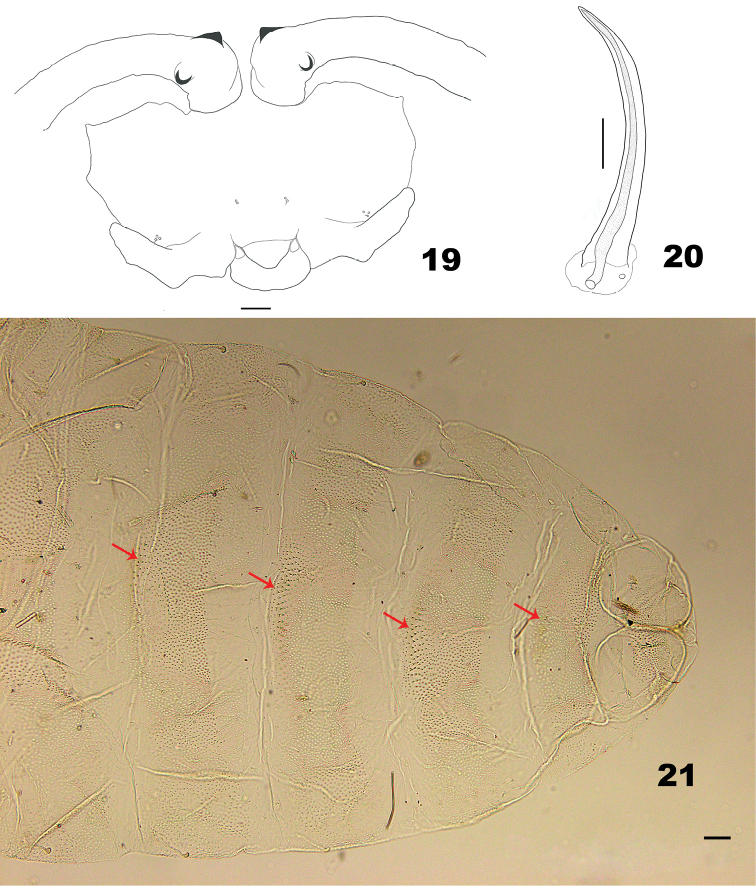
Pupa of *Massalongia nakamuratetsui* sp. nov. **19** ventral view of head **20** prothoracic spiracle **21** dorsal view of terminal part of abdomen (arrows indicate dorsal spines). Scale bars: 50 µm.

*Pupa* (Figs 19–20). Exuviae not pigmented except antennal horns and prothoracic spiracles. Two asetose and 2 setose cephalic papillae present. Prothoracic spiracle long, slightly curved. Abdominal spiracles present on segments II–VI. Abdominal segments I–VII each with 6 dorsal papillae. Dorsal and lateral parts of abdominal segments covered evenly with pointed spinules, diminishing gradually in length and width, except on posterior third.

**Figures 22–24.**
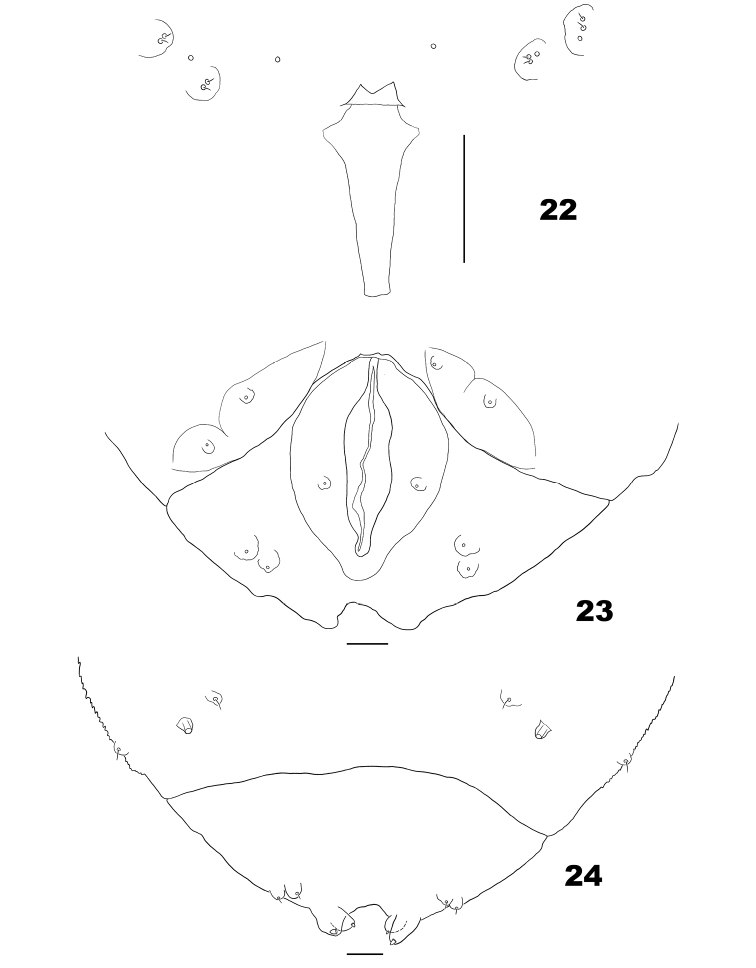
Larva of *Massalongia nakamuratetsui* sp. nov. **22** spatula **23** ventral view of terminal abdominal segments **24** dorsal view of terminal abdominal segments. Scale bars: 50 µm.

*Mature larva*. Spatula bilobed (Fig. 22) or absent (Fig. 39). Ventral papillar pattern basic for Cecidomyiidi (Gagné 1989). One asetose anal papilla present on each side of anal opening; other 2 asetose papillae situated posterolaterally, each on separate plaque (Fig. 23). Six dorsal papillae present on thoracic segments and abdominal segments I–VII; 2 setose dorsal papillae present on abdominal segment VIII.

##### Remarks.

Comparisons with other possibly related genera revealed that the Nearctic genus *Apagodiplosis* Gagné, which contains a single species, *A. papyriferae* (Gagné), fits the definition of *Massalongia* (Gagné, 1973). No differences were found between the two genera (Gagné 1967, 1973). Thus, we synonymize *Apagodiplosis* under *Massalongia* and *M. papyriferae* (Gagné) is a new combination.

### Taxonomic key to species of *Massalongia*

**Table:** 

1	Gonostylus with pointed denticles (e.g. Fig. 33)	**2**
–	Gonostylus with blunt denticles (e.g. Fig. 16)	**3**
2	Aedeagus cylindrical; male hypoproct entire or slightly notched and as long as cerci (Figs 33, 35)	***M. bachmaieri* Möhn, 1958**
–	Aedeagus narrowed at midlength; male hypoproct notched, longer than cerci (based on Fedotova 1991)	***M. altaica* Fedotova, 1990**
3	Male hypoproct entire; larva without spatula and with 4 corniform terminal papillae	***M. betulifolia* Harris, 1974**
–	Male hypoproct bilobed; larva with bilobed spatula and 4 setose and 4 corniform terminal papillae	**4**
4	Aedeagus enlarged apically and longer than hypoproct (Fig. 52)	***M. rubra* (Kieffer, 1890)**
–	Aedeagus cylindrical and shorter than hypoproct	**5**
5	Gonostylus curved distally; ovipositor has dorsal pigmentation on distal 2 thirds of protrusible portion; anterior lobes of larval spatula curved medially	***M. papyriferae* (Gagné, 1967)**
–	Gonostylus not curved distally (Fig. 16); ovipositor has dorsal pigmentation along protrusible portion (Figs 14, 15); anterior lobes of larval spatula directed anteriorly (Fig. 22)	***M. nakamuratetsui* Elsayed & Tokuda, sp. nov.**

#### Massalongia nakamuratetsui

Taxon classificationAnimaliaDipteraCecidomyiidae

Elsayed & Tokuda sp. nov.

8B4FD163-E57D-5E1A-8170-A8C244097CE9

http://zoobank.org/F9C25334-03BF-4BE4-8A56-8FC7BAA0F096

##### Description.

*Head* (Figs 4–9). Eyes separated on vertex by diameter of 0.0–1.25 facets. Frons with 3–9 setae (n = 9). Mouthparts: labrum with 8–10 setae (n = 9); hypopharynx with thick microtrichia on edges; labellum microtrichose, with 4–5 setae (n = 5); palpal segments consecutively longer. Antenna: scape and pedicel microtrichose and with few ventral setae on basal half; flagellomeres III–XII usually with few microtrichia concentrated on base of node; male flagellomere XII sometimes pointed apically.

*Thorax* (Figs 10, 11). Wing 2.6–2.9 mm long in males (n = 6), 3.1–3.3 mm long in females (n = 3). Anepimeral setae 11–17 (n = 9).

*Female abdomen* (Figs 12–15). Tergites I–VII with few lateral setae. Ovipositor: stiff dorsal sclerite present along protrusible portion, posteriorly wider than anteriorly; protrusible portion ca. 1.3 as long as tergite VII; cerci elongated, with scattered setae lateroapically; hypoproct short.

*Male abdomen*. Tergite VIII without posterior row of setae. Terminalia (Figs 16–18): gonostylus with blunt denticles, ventrally with cluster of short setae near base; cerci with tapered and setose apex, basal part of cerci without setae; hypoproct bilobed, narrowed after midlength; aedeagus shorter than cerci and hypoproct, cylindrical in dorsoventral view, wide basally in lateral view.

*Pupa* (Figs 19–21). Antennal horns with short, acute, apical protuberances; 2 setose and 2 asetose lower facial papillae present; 1 asetose and 2 setose lateral facial papillae present on each side. Prothoracic spiracle, about 270 μm long, with trachea extending to tip. Abdominal terga I–VII each with 4 setose and 2 asetose dorsal papillae; terga VIII with 4 setose dorsal papillae. Abdominal terga II–VIII with 3–4 median rows of slightly wider and longer spinules than surrounding ones.

*Mature larva* (Figs 22–24). In life, orange. Spatula short and bilobed. Dorsal papillae without setae on thoracic segments, with setae on abdominal segments. Two asetose pleural papillae on thoracic segments; 2 setose and 1 asetose pleural papillae on abdominal segments. Terminal segment with 8 papillae: 4 corniform and 4 setose.

##### Etymology.

The species is named in honor of the late Japanese physician Dr. Tetsu Nakamura in recognition to his lifelong dedication to supporting poor people and his significant contributions to the development of Afghanistan. Dr. T. Nakamura was fatally shot by extremists on 4 December 2019 in Afghanistan, exactly on the date when we prepared the first draft of this paper and were considering what to name the species. In this way, we wish to immortalize his contributions to humanity.

##### Type material.

***Holotype***. 1♂ (ELKU): Reared from larvae in bottle-like cocoons collected under *B. grossa* by A. K. Elsayed on 15.xii.2018 from Mount Tara, Saga Prefecture, Japan on 15.xii.2018, emerged on 15.iii.2019. ***Paratypes***. All reared from larvae in bottle-like cocoons collected under *B. grossa* by A. K. Elsayed at the type locality, as follows. 3 larvae: obtained from cocoons on 15.xii.2018; 1 pupal exuviae: emerged on 23.iii.2019; 2♂, 1♀, 2 pupal exuviae: emerged on 27.iii.2019; 2♂, 2♀: emerged on 30.iii.2019; 1♂: emerged on 4.iv.2019.

##### DNA accession numbers.

LC557490–LC557493.

##### Distribution.

Japan: Kyushu Island, Saga Prefecture.

##### Gall and life history.

*Massalongia nakamuratetsui* forms galls on the midveins of *B. grossa* (Fig. 1). One leaf can bear several galls and some galls become fused with larvae occupying separate chambers. Galls are 1.52–3.10 mm in diameter and 6.46–18.03 mm long. Galls collected at the end of August contained white first instars. Larvae develop to second and mature larvae by the end of September. In late October, the mature larvae leave the galls to overwinter in the ground, where they spin hyaline, bottle-shaped cocoons on leaf litter (Figs 2, 3). The cocoon of *M. nakamuratetsui* is waterproof and does not allow water to reach the overwintering larva (Suppl. material 1: Video S1). Adults emerge between the end of March and the beginning of April.

##### Remarks.

*Massalongia nakamuratetsui* is most similar to *M. papyriferae*, sharing a bilobed sternal spatula, four setose and four coniform larval terminal papillae, gonostyli ending with blunt denticles and bilobed male hypoproct (Gagné 1967, 1973). They can be separated as follows: anterior lobes of spatula are directed anteriorly in *M. nakamuratetsui*, but curved toward each other in *M. papyriferae*; gonostylus is less curved distally in *M. nakamuratetsui* compared to *M. papyriferae*; ovipositor has dorsal pigmentation along the protrusible portion in *M. nakamuratetsui*, but only on the distal two thirds in *M. papyriferae*.

#### Massalongia bachmaieri

Taxon classificationAnimaliaDipteraCecidomyiidae

Möhn, 1958

78ED4129-D052-5C7B-8EC9-FAED86F2159C

##### Description.

**Figures 25–29.**
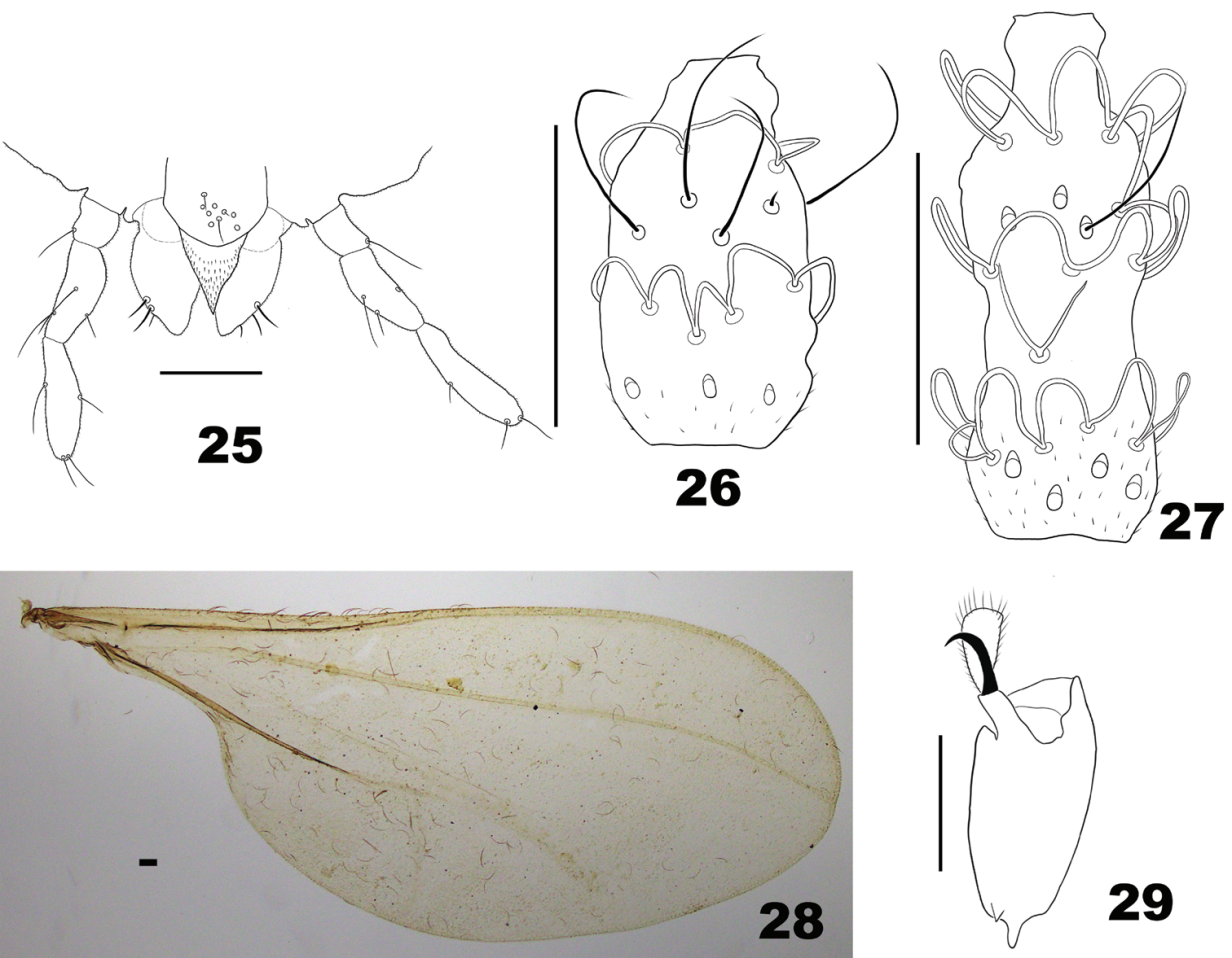
*Massalongia bachmaieri*. **25** ventral view of mouthparts **26** ventral view of female flagellomere V. **27** ventral view of male flagellomere III **28** wing **29** tarsomere V and acromere. Scale bars: 50 µm.

*Head* (Figs 25–27). Eyes separated on vertex by diameter of 0.0–1.5 facets. Frons with 3–9 setae (n = 6). Mouthparts: labrum with 8–17 short setae (n = 9), hypopharynx pointed, mostly microtrichose; labellum with 4–5 stout setae (n = 5) laterally. Antenna: scape and pedicel with few ventral setae.

*Thorax* (Figs 28, 29). Wing 2.10–2.25 mm long in males (n = 4), 2.10–2.30 mm long in females (n = 4). Anepimeral setae 3–6 (n = 6).

**Figures 30–32.**
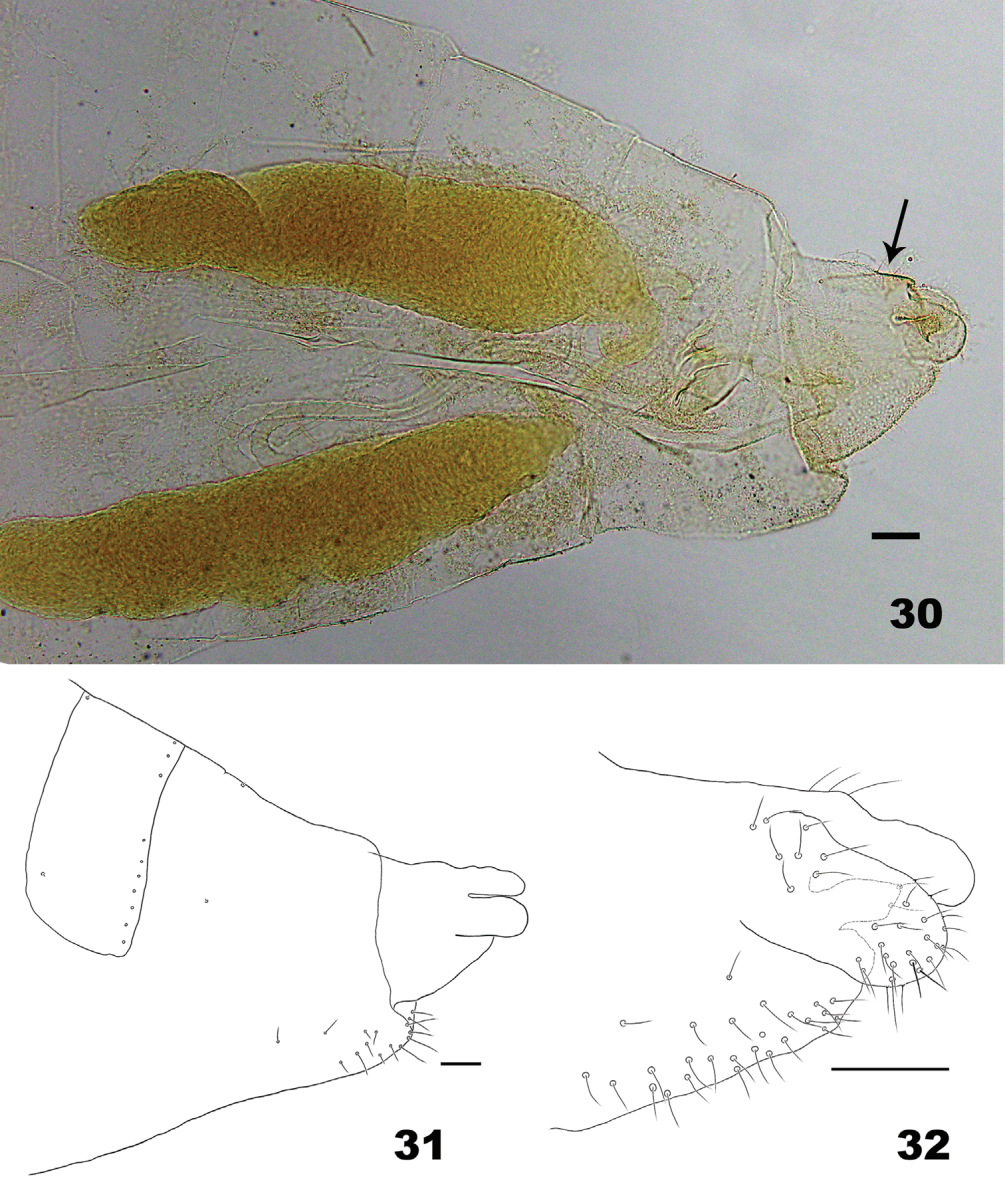
*Massalongia bachmaieri.***30–31** terminal part of female abdomen (arrow in Fig. 31 indicate the dorsal sclerite on the protrusible portion) **32** protrusible portion of ovipositor. Scale bars: 50 µm.

*Female abdomen* (Figs 30–32). Ovipositor: protrusible portion ca. 1.2 as long as tergite VII, with dorsal sclerite on posterior 2 thirds; cerci elongate-ovoid, with dorsal setae on base, and scattered setae apically.

**Figures 33–35.**
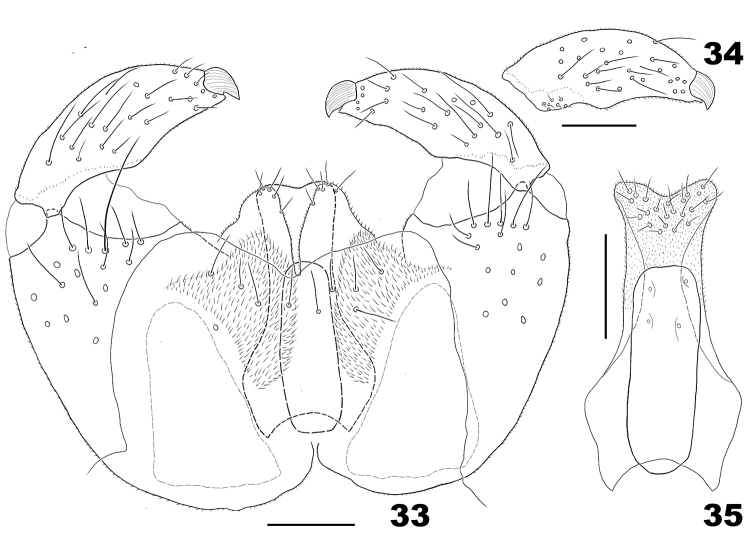
*Massalongia bachmaieri*. **33** male terminalia **34** ventral view of gonostylus **35** ventral view of male hypoproct and aedeagus. Scale bars: 50 µm.

*Male abdomen*. Tergite VIII with posterior row of setae. Terminalia (Figs 33–35): gonostylus with pointed denticles; cerci base with setae; cerci with setae on apical margin; hypoproct entire, slightly notched, narrowed after basal third; aedeagus shorter than cerci and hypoproct, cylindrical in dorsoventral view, wide basally in lateral view.

**Figures 36–38.**
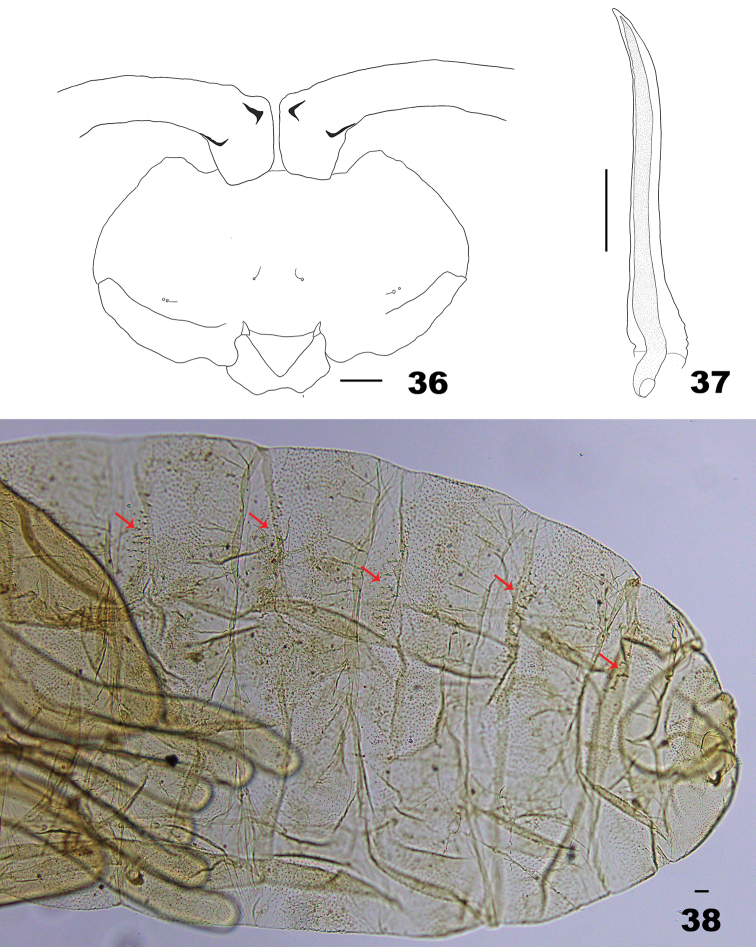
Pupa of *Massalongia bachmaieri*. **36** ventral view of head **37** prothoracic spiracle **38** dorsal view of terminal part of abdomen (arrows indicate dorsal spines). Scale bars: 50 µm.

*Pupa* (Figs 36–38). Head and thorax of exuviae slightly pigmented; abdomen not pigmented. Antennal horns with short, acute, apical protuberances. Two setose lower facial papillae present; 1 asetose and 1 setose lateral facial papillae present on each side. Prothoracic spiracle long, ca. 210 μm, with trachea extending to just before tip. Abdominal segment VIII with 2 setose dorsal papillae. Abdominal terga II–VIII with 2–3 median rows of wider and longer spinules than surrounding ones.

**Figures 39–41.**
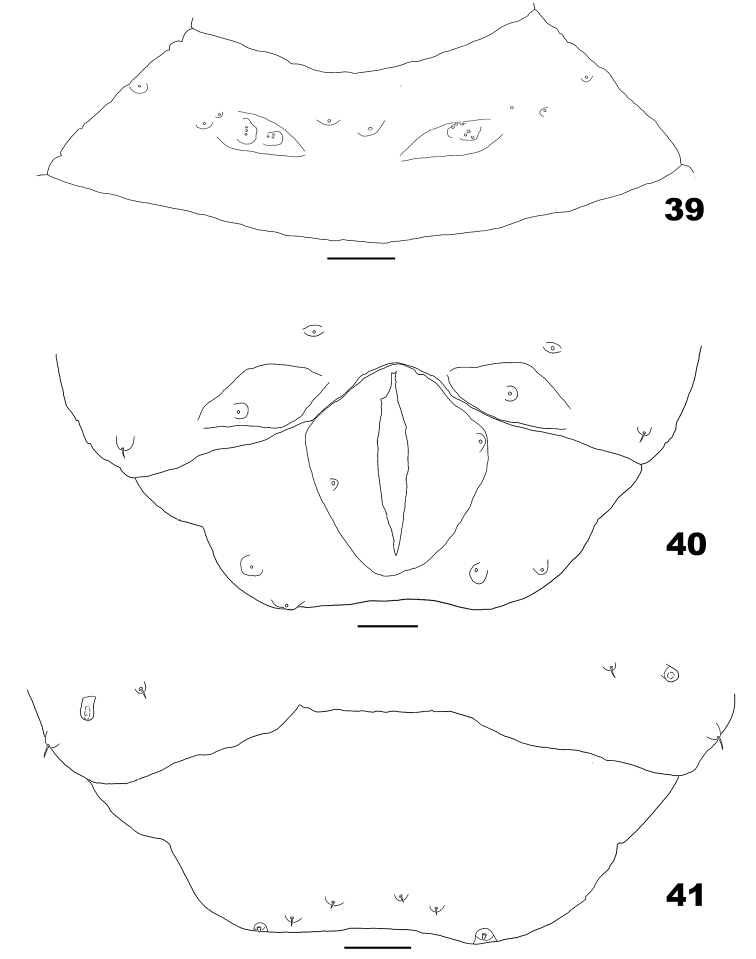
Larva of *Massalongia bachmaieri*. **39** ventral view of prothoracic segment **40** ventral view of terminal abdominal segments **41** dorsal view of terminal abdominal segments. Scale bars: 50 µm.

*Mature larva* (Figs 39–41). Orange to red (Bachimaier 1965). Spatula absent. Terminal segment with 6 papillae: 2 tiny corniform and 4 setose papillae.

##### Material examined.

*Lectotype*. 1♂ (SMNS): obtained from Möhn collection Nr. 1205. *Paralectotypes*. 8 larvae collected on 10.iv.1954 from leaf galls on *B. nana*, Bernrieder Filz; 3♂, 4♀, 2 pupal exuviae in Möhn collection Nr. 1205.

##### Distribution.

Europe: Germany and Russia (Gagné and Jaschhof 2017).

##### Gall and life history.

**Figure 42.**
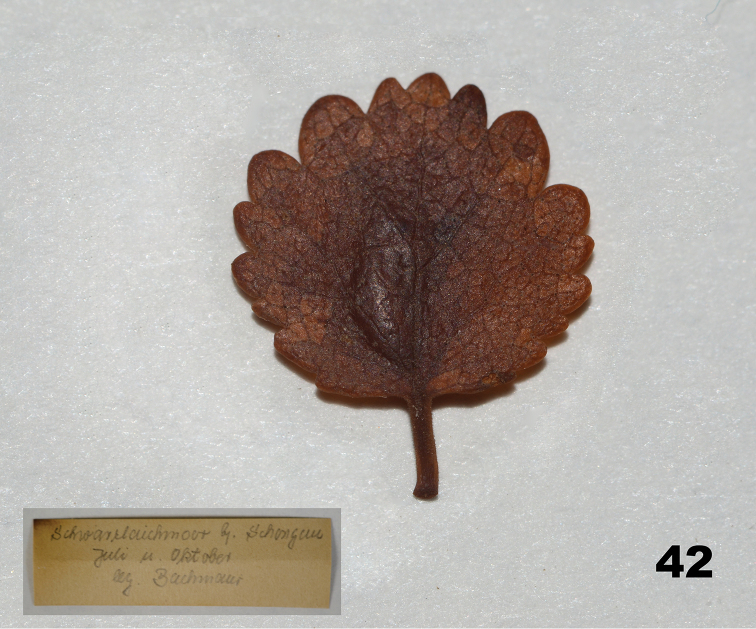
Ethanol-preserved leaf gall of *Massalongia bachmaieri* on *B. nana*.

*Massalongia bachmaieri* induces parenchymal leaf galls on *B. nana* (Fig. 42). Mature larvae leave the galls and drop to the ground in mid to late October. They overwinter in cocoons that are spun on the fallen leaves. This species has one generation a year (Möhn 1958; Bachimaier 1965).

##### Remarks.

Möhn (1958) designated a male specimen as a holotype of *M. bachmaieri* and two males and a female as paratypes. When we requested the types for this study, we found that all specimens deposited in SMNS were preserved in alcohol. Möhn probably prepared his illustrations of the species from temporary slide mounts and then put the specimens back in alcohol with the others. Because it was not possible to determine Möhn’s holotype and paratypes among these ethanol-preserved specimens, we designated a lectotype and paralectotypes from the permeant slide-mounted specimens we prepared.

Adults of *M. bachmaieri* are morphologically most similar to *M. altaica*. See more under *M. altaica* below.

#### Massalongia betulifolia

Taxon classificationAnimaliaDipteraCecidomyiidae

Harris, 1974

82DFBD0D-FE30-5902-B7B0-A0D4E7A6D34D

##### Description.

**Figures 43–47.**
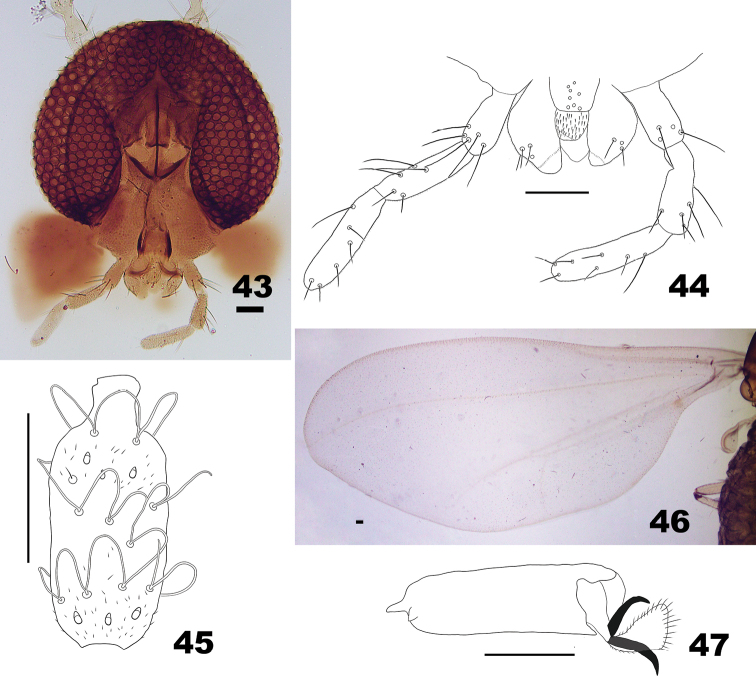
*Massalongia betulifolia*. **43** head **44** ventral view of mouthparts (hypopharynx is folded). **45** dorsal view of male flagellomere VIII **46** wing **47** tarsomere V and acromere. Scale bars: 50 µm.

*Head* (Figs 43–45). Eyes separated on vertex by diameter of 0.5–1.25 facets. Frons with 7–12 setae (n = 5). Mouthparts: labrum with 6–11 short setae (n = 5), hypopharynx pointed, covered with thick microtrichia; labellum with 5–8 stout setae (n = 4) laterally. Antenna: scape and pedicel with few ventral setae on basal third of segment.

*Thorax* (Figs 46, 47). Wing 2.8–3.0 mm long in males (n = 2), 3.1–3.2 mm in females (n = 2). Anepimeral setae 6–10 (n = 6).

**Figures 48–49.**
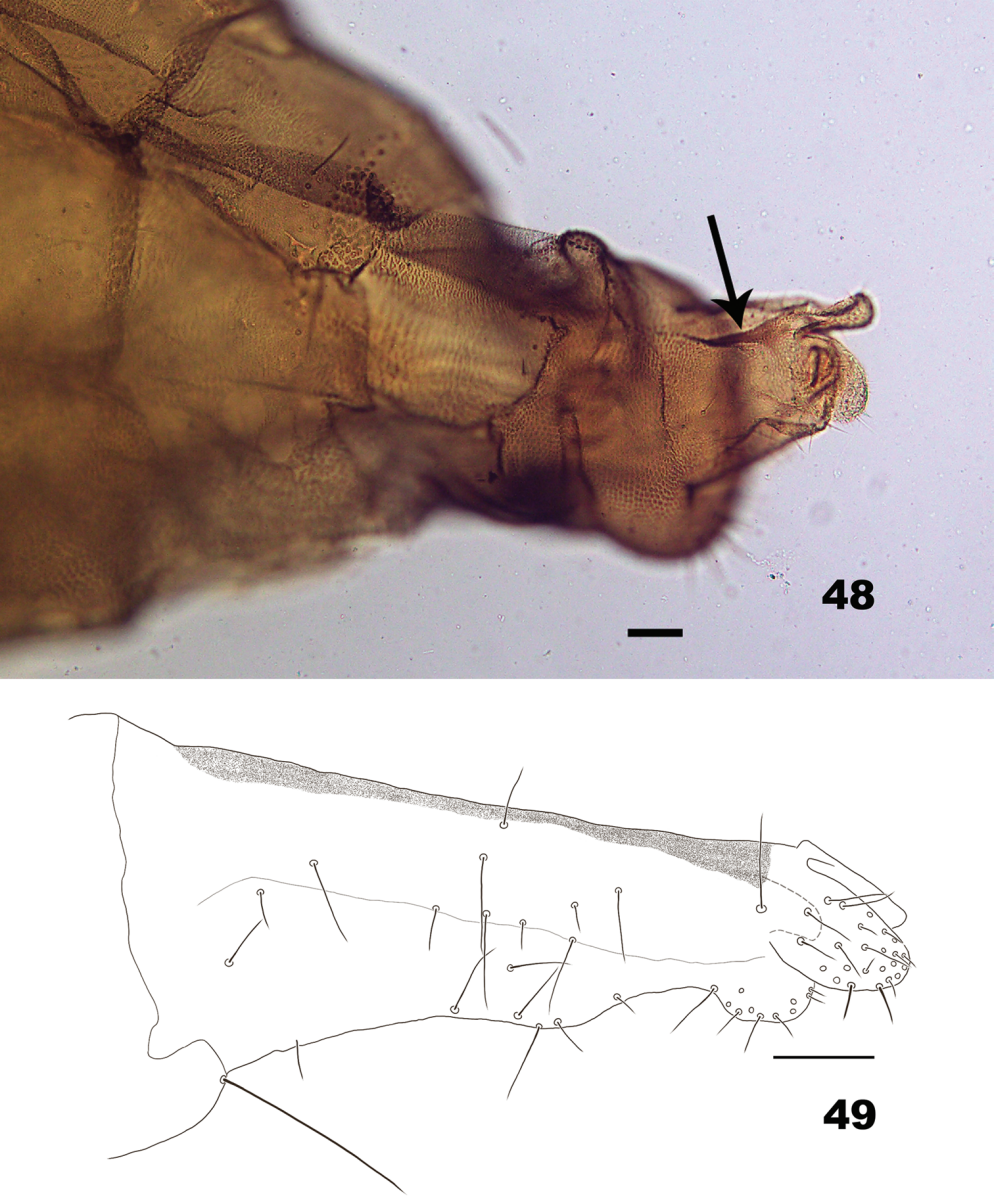
*Massalongia betulifolia.***48** terminal part of female abdomen (arrow indicate the dorsal sclerite on the protrusible portion) **49** protrusible portion of ovipositor. Scale bars: 50 µm.

*Female abdomen* (Figs 48, 49). Tergites I–VI bare posteromedially; tergite VII with entire posterior row of setae. Ovipositor: protrusible portion with, ca. 2 times as long as tergite VII, with dorsal sclerite almost along dorsal portion; cerci setose.

**Figures 50–51.**
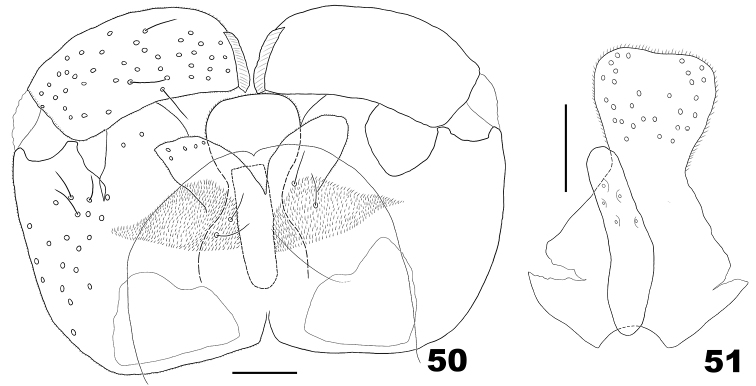
*Massalongia betulifolia*. **50** male terminalia **51** ventral view of male hypoproct and aedeagus. Scale bars: 50 µm.

*Male abdomen*. Tergites I–VII as in female; tergite VIII with few setae posteriorly. Terminalia (Figs 50, 51): gonostylus with blunt denticles; cerci base with few setae; cerci with setae apically; hypoproct entire, narrowed at midlength; aedeagus shorter than cerci and hypoproct, cylindrical in dorsoventral view, wide basally in lateral view.

*Mature larva*. Spatula absent. Terminal segment with short conical papillae (Harris 1974).

*Pupa*. Exuviae generally unpigmented. Antennal horns short (Askew and Ruse 1974).

**Materials examined. *Holotype*** (BMNH): 1♂, emerged on vi.1971, reared by J. Ruse from larva forming lamina and midrib blister galls on *Betula pendula* leaves, collected on x.1970 from Lindow Common, Cheshire, England. ***Paratypes***: 4♂, 2♀, data same as for holotype.

##### Distribution.

Europe: England and Norway (Gagné and Jaschhof 2017).

##### Gall and life history.

*Massalongia betulifolia* forms blister-like leaf galls on *B. pendula* and *B. pubescens*. Galls are formed usually between or on veins and are 2.5–3.0 mm wide and 5.0–6.0 mm long. Mature larvae drop to the ground to overwinter in cocoons. Adults emerge probably in May and June, and the galls can be found on the trees between June to October (Harris 1974; Askew and Ruse 1974).

##### Remarks.

See Remarks under *M. bachmaieri* and *M. nakamuratetsui*.

#### Massalongia rubra

Taxon classificationAnimaliaDipteraCecidomyiidae

(Kieffer, 1890)

403E4701-F4F6-51F4-A384-42BD73A8C854

Hormomyia rubra Kieffer, 1890: 199.
Oligotrophus ruber Kieffer, 1895: lxxi.

##### Description.

*Female abdomen*. Ovipositor: protrusible portion long; cerci elongated; hypoproct short (Kieffer 1913b).

**Figures 52–54.**
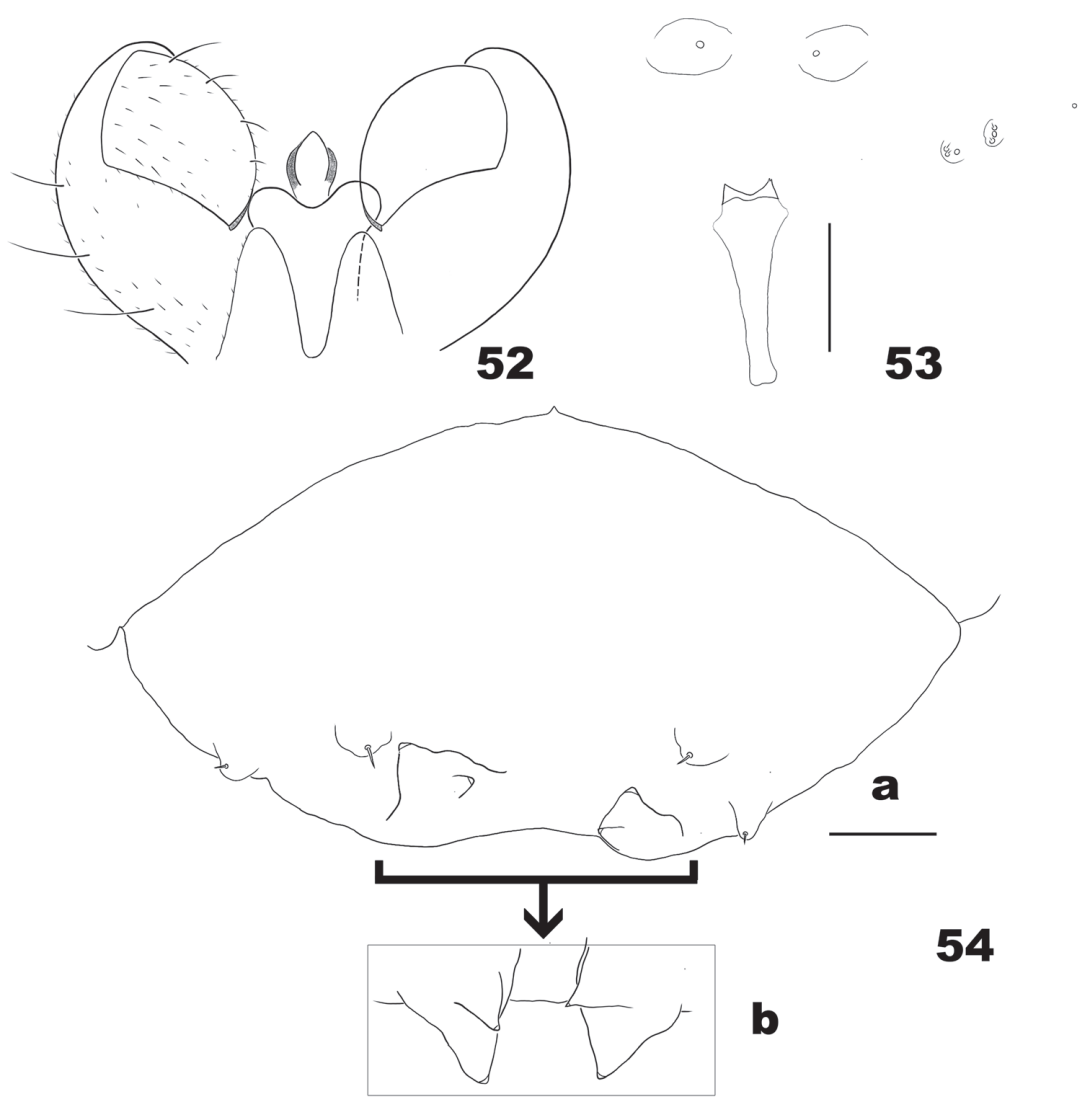
*Massalongia rubra*. **52** male terminalia (after Kieffer 1913b) **53** spatula **54** dorsal view of larval terminal abdominal segments (**a** the inner four terminal papillae are folded on the segment **b** the inner four terminal papillae are unfolded in another specimen). Scale bars: 50 µm.

*Male abdomen*. Terminalia: gonostyli with blunt denticles; cerci with rounded tips, shorter than hypoproct; hypoproct notched; aedeagus longer than hypoproct, with enlarged tip (Fig. 52) (Kieffer 1913b).

*Mature larva* (Figs 53, 54). Spatula bilobed. Dorsal papillae on thoracic segments with tiny setae. Terminal segment with 4 corniform papillae, outer 2 longer than inner ones, and 2 setose papillae.

*Pupa*. Unknown.

##### Material examined.

3 larvae: collected in August 1964 from *Betula* sp. Pěčín Village, Rychnov nad Kněžnou Region, Hradec Králové, Czech Republic, M. Skuhravá leg.

##### Distribution.

Widespread in Europe and west Asia (Gagné and Jaschhof 2017).

##### Gall and life history.

*Massalongia rubra* induces barely noticeable midrib leaf galls on *Betula pubescens* Ehrh. and other *Betula* spp. (Gagné and Jaschhof 2017; Kieffer 1913b). The females lay eggs on young leaves in May, and most mature larvae leave the galls to overwinter in the ground in October, but some hibernate in the galls (Skuhravá and Skuhravý 1973).

##### Remarks.

The larval specimens we described here were collected from similar galls to those described by Kieffer (1913b) for *M. rubra* and the larval morphology fits Kieffer’s description and illustrations, thus we believe they indeed belong to *M. rubra*. Types of *M. rubra*, like most of Kieffer’s types, are considered lost (Gagné and Jaschhof 2017). We considered using one larva for designating a neotype for the species, but because no adults were reared from these larvae, we cannot be completely certain about their identity and decided to refrain from doing so. Kieffer (1913b) provided an illustration of male terminalia showing that the species is distinctive from the other known species of *Massalongia* by its long and apically enlarged aedeagus (Fig. 52). Because this illustration is important for separating species and it was drawn from the type specimen, future designation of a neotype for the species must rely on reared adults that will enable to compare characters of the male terminalia.

#### Massalongia papyriferae

Taxon classificationAnimaliaDipteraCecidomyiidae

(Gagné, 1967) comb. nov.

CE30A491-EF5D-52AE-9718-C8DA591C651A

Oligotrophus papyriferae Gagné, 1967: 132.
Apagodiplosis papyriferae Gagné, 1973: 862.

##### Description.

(Based on Gagné 1967, 1973). *Head*. Frons with 2–5 setae. Male flagellomere XII tapered distally.

*Thorax*. Anepimeron with 7–17 setae.

*Female abdomen*. Tergites I–VI bare posteromedially; tergite VII with entire posterior row of setae. Ovipositor: protrusible portion with pencil-shaped dorsal sclerite on posterior 2 thirds.

*Male abdomen*. Terminalia: gonostyli with blunt denticles; cerci rounded; hypoproct bilobed; aedeagus shorter than cerci and hypoproct.

*Pupa*. Antennal horns short; 2 setose lower facial papillae present; cephalic setae short. Abdominal terga covered with uniformly tiny spinules.

*Larva*. Spatula short, bilobed, with anterior lobes slightly curved toward each other. Terminal segment with 4 corniform papillae and 4 setose terminal papillae.

##### Distribution.

North America: Canada (Quebec) and USA (Washington, Oregon and Michigan) (Gagné and Jaschhof 2017).

##### Gall and life history.

*Massalongia papyriferae* forms bud galls on the paper birch, *B. papyrifera*. The mature larva drops to the leaf litter to overwinter in a bottle-shaped cocoon. Adults emerge in spring (Gagné 1967).

##### Remarks.

See under *M. nakamuratetsui*.

#### Massalongia altaica

Taxon classificationAnimaliaDipteraCecidomyiidae

Fedotova, 1990

15028DC7-A5D8-576C-B926-4268E5F0DAFC

##### Description.

(Based on Fedotova 1991). *Head*. Female flagellomere XII variable in shape, rounded to oblong with apical constriction; male flagellomere XII slightly tapered distally.

*Thorax*. Wing length 2.7 times width.

*Female abdomen*. Ovipositor: cerci rounded, microtrichose, without setae.

*Male abdomen*. Terminalia: gonostylus with pointed denticles; cerci triangular; hypoproct bilobed, longer than cerci; aedeagus widened apically and basally, shorter than hypoproct.

*Pupa*. Unknown.

*Larva*. Unknown.

##### Distribution.

Kazakhstan: Central Altaï, Koksuïskiï Mountain Range, Lymin Belok Mt., 60 km NE of Leninogorsk (Fedotova 1991).

##### Gall and life history.

*Massalongia altaica* form barely visible swellings, 5–7 mm long, on the leaves of Betula nana var. rotundifolia (Spach) Regel. (*B. rotundifolia* in the original description). The mature larva leaves the gall through an opening on the lower side of the leaf and overwinters in the ground (Fedotova 1991).

##### Remarks.

*Massalongia altaica* was described from adult specimens reared from larvae that emerged from leaf galls on *B. rotundifolia*, which is currently known as a variety of *B. nana*, the same host plant of *M. bachmaieri* (The Plant List 2013). The illustration of *M. altaica* galls provided in its original description (Fedotova 1991) is quite similar to the galls of *M. bachmaieri* (Fig. 43). Morphologically, the adults of *M. altaica* are closest to *M. bachmaieri* and differ from them only in the shape of the aedeagus and male hypoproct and the relative length of cerci to male hypoproct, but these differences are based on the original description of *M. altaica* (Fedotova 1991). Because the type specimens of *M. altaica* were not available to us, we could not verify the differences between *M. altaica* and *M. bachmaieri*. A future examination of *M. altaica* types and its immature stages may result in synonymizing it under *M. bachmaieri*.

## Discussion

*Massalongia* has been considered so far a Palearctic genus (Gagné and Jaschhof 2017), but in the present study we synonymized the Nearctic *Apagodiplosis* under *Massalongia*, thus the distribution of *Massalongia* corresponds now to that of its Holarctic host plant, *Betula* (Shaw et al. 2014). Comparing the sequence data of *M. nakamuratetsui* with all sequences available in The Barcode of Life Data (BOLD) system revealed several sequences with interspecific similarity of up to 96.85% (Ratnasingham and Hebert 2007), all from Canada (Hebert et al. 2016). The profile of one of these cecidomyiids (sequence ID: CNPKE263–14) included a photo of a female specimen that resembles *Massalongia* and the interspecific similarity was 95.3%. This strongly supports *Massalongia* as a Holarctic genus and suggests that more *Massalongia* species can be discovered in the Nearctic region.

Larvae of many gall midge species that drop to the ground are known to spin cocoons in which they overwinter and eventually pupate (Gagné 1989). Bakhshi and Grover (1976) studied cocoons of various gall midge taxa and concluded that the cocoon shape is specific to genus. The bottle-shaped cocoon of *Massalongia* has never been reported from other gall midge taxa and thus it appears to be a unique characteristic of the genus. Cocoons of many insects that overwinter in the soil provide mechanical protection against unfavorable surrounding conditions (Danks 2004). Because the cocoon of *M. nakamuratetsui* is waterproof, the bottle-like cocoons of *Massalongia* possibly represent a protective adaptation for pupation in wet and snowy lands. Further research on these cocoons is necessary in order to understand the nature of its texture and other roles of its bottle-like shape.

## Supplementary Material

XML Treatment for Massalongia

XML Treatment for Massalongia nakamuratetsui

XML Treatment for Massalongia bachmaieri

XML Treatment for Massalongia betulifolia

XML Treatment for Massalongia rubra

XML Treatment for Massalongia papyriferae

XML Treatment for Massalongia altaica

## Supplementary materials

Supplementary material 1 Video S1 Data type multimedia https://binary.pensoft.net/file/440979This dataset is made available under the Open Database License (http://opendatacommons.org/licenses/odbl/1.0/). The Open Database License (ODbL) is a license agreement intended to allow users to freely share, modify, and use this Dataset while maintaining this same freedom for others, provided that the original source and author(s) are credited.Ayman Khamis Elsayed, Marcela Skuhravá, Kazuki Ohta, Satoshi Yoshida, Makoto Tokuda
